# Development and Internal Validation of a Preintubation Prediction Model for Postintubation Hypotension in Trauma Patients Undergoing Emergency Endotracheal Intubation: A Retrospective Cohort Study

**DOI:** 10.3390/jcm15208102

**Published:** 2026-10-19

**Authors:** Chanatthee Kitsiripant, Komet Thongkhao, Osaree Akaraborworn, Siriwimon Tantarattanapong

**Affiliations:** 1 Department of Anesthesiology, Faculty of Medicine, Prince of Songkla University, Hat Yai 90110, Thailand; 2 Division of Trauma and Critical Care, Department of Surgery, Faculty of Medicine, Prince of Songkla University, Hat Yai 90110, Thailand; komet014@hotmail.com (K.T.); aosaree@gmail.com (O.A.); 3 Department of Emergency Medicine, Faculty of Medicine, Prince of Songkla University, Hat Yai 90110, Thailand; tsiriwimon.er@gmail.com

**Keywords:** airway management, emergency department, postintubation hypotension, prediction model, emergency endotracheal intubation, trauma

## Abstract

**Background/Objectives:** Postintubation hypotension (PIH) is an important complication of emergency endotracheal intubation in trauma patients. However, the applicability of preintubation predictors of PIH in trauma patients undergoing emergency endotracheal intubation in the emergency department (ED) remains uncertain. Here, we aimed to develop and internally validate a preintubation prediction model for PIH in such patients. **Methods:** This retrospective cohort included trauma patients aged ≥ 15 years undergoing emergency endotracheal intubation in the ED during 2015–2023. PIH was defined as systolic blood pressure < 90 mmHg within 30 min after intubation. Eight preintubation candidate predictors were evaluated using logistic regression with backward elimination. Internal validation used 2000 bootstrap resamples repeating the entire variable-selection procedure. **Results:** Among 348 patients, 60 (17.2%) developed PIH; 345 patients with 57 PIH events had complete data for model development. The final model retained systolic blood pressure, body temperature, hematocrit, and torso injury, with adjusted odds ratios of 1.19 per 10-mmHg lower systolic blood pressure, 1.48 per 1 °C lower temperature, 1.33 per 5-percentage-point lower hematocrit, and 3.26 for torso injury. The apparent and optimism-corrected areas under the receiver operating characteristic curve were 0.742 and 0.700, respectively. **Conclusions:** A four-variable preintubation prediction model demonstrated modest optimism-corrected discrimination and substantial model-selection uncertainty. External validation and assessment of clinical utility are required before clinical implementation.

## 1. Introduction

Emergency endotracheal intubation is a critical intervention in trauma care, providing definitive airway control in patients with airway compromise, hypoxemia, or severe traumatic brain injury [1,2]. However, emergency airway management can impose substantial physiological stress, particularly in patients with occult hemorrhage, hypovolemia, or impaired cardiovascular reserve. The combined effects of induction agents and the transition to positive-pressure ventilation can precipitate acute hemodynamic deterioration [1,3]. Postintubation hypotension (PIH) is an important complication of emergency airway management and is associated with cardiac arrest, greater critical care utilization, and mortality [3,4,5]. PIH is independently associated with increased mortality among trauma patients, underscoring the importance of maintaining hemodynamic stability during emergency airway management [5].

Early identification of patients at risk of PIH may allow clinicians to anticipate hemodynamic deterioration and optimize resuscitation before airway intervention. Previous studies have identified preintubation physiological predictors of PIH and developed prediction approaches in emergency department (ED) and prehospital settings [6,7,8,9]. However, their applicability to trauma patients undergoing emergency endotracheal intubation in the ED remains uncertain because of differences in patient populations, clinical settings, predictor availability, resuscitation practices, and airway management protocols. Trauma-specific evidence from Asian ED populations also remains limited.

A pragmatic prediction model based on information obtained before intubation could provide a structured approach to PIH risk assessment without relying on procedural or postintubation information. To address this gap, we aimed to develop and internally validate a preintubation prediction model for PIH in trauma patients undergoing emergency endotracheal intubation in the ED.

## 2. Materials and Methods

### 2.1. Study Design, Setting, and Participants

This retrospective cohort study was conducted at Songklanagarind Hospital, a university-affiliated level I trauma center in southern Thailand. Consecutive trauma patients aged ≥ 15 years who underwent emergency endotracheal intubation in the ED between 1 January 2015 and 31 December 2023 were screened for eligibility. Patients who had undergone endotracheal intubation before ED arrival, experienced cardiac arrest before intubation, or had incomplete records precluding determination of the primary outcome were excluded. Eligible patients were identified from the institutional trauma registry and cross-referenced with electronic medical records.

During the study period, emergency endotracheal intubation was performed according to the patient’s clinical condition and the treating physician’s judgment, without a standardized medication protocol. The selection of induction agents and use of neuromuscular blocking agents varied according to clinical circumstances and routine emergency department practices.

The study is reported in accordance with the Transparent Reporting of a Multivariable Prediction Model for Individual Prognosis or Diagnosis plus Artificial Intelligence (TRIPOD + AI) statement [10].

### 2.2. Outcomes

The primary outcome for prediction-model development was PIH, defined as any recorded systolic blood pressure (SBP) < 90 mmHg within the first 30 min after successful endotracheal intubation. Blood pressure was measured noninvasively in the ED at 15–30-min intervals as part of routine clinical monitoring. PIH was ascertained from all available blood pressure measurements recorded during the 30-min postintubation period.

### 2.3. Candidate Predictors

Demographic characteristics, injury-related variables, preintubation physiological measurements, and laboratory findings were obtained from the institutional trauma registry and electronic medical records using a standardized data collection form. For physiological variables, the last measurements recorded before emergency endotracheal intubation were used. At our institution, body temperature was routinely measured during initial ED triage. Trauma patients requiring resuscitation were subsequently managed in the ED resuscitation area, where intravenous access was promptly established and blood samples for laboratory investigations, including hematocrit, were generally collected during initial resuscitation.

Variables collected included age, sex, body mass index, comorbidities, mechanism and anatomical distribution of injury, preintubation SBP, diastolic blood pressure (DBP), heart rate, respiratory rate, peripheral oxygen saturation, body temperature, Glasgow Coma Scale (GCS) score, hematocrit, and Injury Severity Score (ISS). Torso injury was defined as documented injury involving at least one of the thoracic, abdominal, or pelvic regions.

Candidate predictors were selected based on clinical relevance, biological plausibility, previously reported associations with peri-intubation hemodynamic deterioration, and availability at the intended time of prediction. Eight preintubation variables were considered: SBP, DBP, heart rate, body temperature, GCS score, mechanism of injury, torso injury, and hematocrit. ISS and the number of injured anatomical regions were not considered candidate predictors because they require more comprehensive injury assessment and may not be reliably available before emergency intubation.

The intended prediction time point was immediately before emergency endotracheal intubation. Accordingly, candidate predictors were restricted to clinical information obtained before intubation to facilitate preintubation risk assessment. Variables reflecting airway management or treatment during or after intubation, including induction agents, neuromuscular blocking agents, airway devices, number of intubation attempts, vasopressor administration, and postintubation physiological measurements, were not considered candidate predictors. Although these factors may influence the occurrence of PIH, their inclusion would change the intended application of the model from preintubation risk prediction to peri-intubation assessment and, for variables determined after intubation, could introduce temporal information leakage.

### 2.4. Sample Size and Model Complexity

All eligible patients meeting the study criteria during the study period were included, and no additional sampling was performed. The study cohort comprised 348 patients, of whom 60 developed PIH. Preintubation body temperature was missing for three patients, all of whom developed PIH. Because missing data were not imputed, model development was based on 345 complete cases, including 57 PIH events. Eight clinically selected candidate predictors were evaluated. The limited number of outcome events relative to the number of candidate predictors was considered a potential source of model overfitting and was addressed in the assessment of model performance through bootstrap internal validation that repeated the entire model-development procedure.

### 2.5. Statistical Analysis

Continuous variables are presented as means with standard deviations or medians with interquartile ranges (IQRs), according to their distributions. Categorical variables are presented as frequencies and percentages. Baseline characteristics were compared between patients with and without PIH using Student’s t-test or the Wilcoxon rank-sum test for continuous variables and the chi-squared test or Fisher’s exact test for categorical variables, as appropriate.

For prediction-model development, preintubation SBP, DBP, heart rate, body temperature, GCS score, and hematocrit were retained on their original continuous scales to avoid loss of predictive information from dichotomization. Mechanism of injury was modeled as blunt versus nonblunt trauma, and torso injury was modeled as a binary variable. All eight candidate predictors were entered into the initial multivariable logistic regression model.

Given the limited number of outcome events, backward elimination was used as an exploratory approach to derive a parsimonious model from the eight prespecified candidate predictors. At each step, the predictor with the highest *p*-value was removed until all retained predictors had a two-sided *p*-value < 0.05. Adjusted odds ratios (aORs) with 95% confidence intervals (CIs) were reported on clinically interpretable scales: per 10-mmHg decrease in SBP, per 1 °C decrease in body temperature, per 5-percentage-point decrease in hematocrit, and presence versus absence of torso injury.

Model discrimination was quantified using the area under the receiver operating characteristic curve (AUROC), and overall predictive accuracy was assessed using the Brier score. Apparent model performance was estimated in the development dataset. A 95% CI for the apparent AUROC was estimated using bootstrap resampling. The final model was retained in its continuous form; no point-based score or clinical decision threshold was derived.

Internal validation was performed using 2000 bootstrap resamples. In each bootstrap resample, the entire model-development procedure was repeated, beginning with all eight candidate predictors and applying the same backward-elimination procedure. Each bootstrap-derived model was evaluated in both the bootstrap sample and original development dataset, and the average difference in performance was used to estimate optimism. Optimism-corrected AUROC and Brier score were obtained by subtracting the estimated optimism from the corresponding apparent performance. Predictor-selection frequencies across bootstrap resamples were summarized to assess model-selection stability. Calibration was assessed using the mean calibration slope obtained by applying the bootstrap-derived models to the original development dataset. Apparent calibration of the final model was displayed graphically in the development cohort by comparing observed event proportions with mean predicted probabilities across groups of predicted risk. The graphical assessment was not based on an independent validation cohort.

Initial data management and descriptive analyses were performed using Stata version 16.0 (StataCorp LLC, College Station, TX, USA). Prediction-model development, bootstrap internal validation, and final statistical analyses were performed using Python version 3.15.0 (Python Software Foundation, Wilmington, DE, USA), with the statsmodels (version 0.15.0) and scikit-learn (version 1.9.1) packages. All statistical tests were two-sided, with *p* < 0.05 used as the criterion for variable retention during backward elimination and for conventional statistical inference where applicable.

## 3. Results

### 3.1. Patient Characteristics

**Figure 1:**
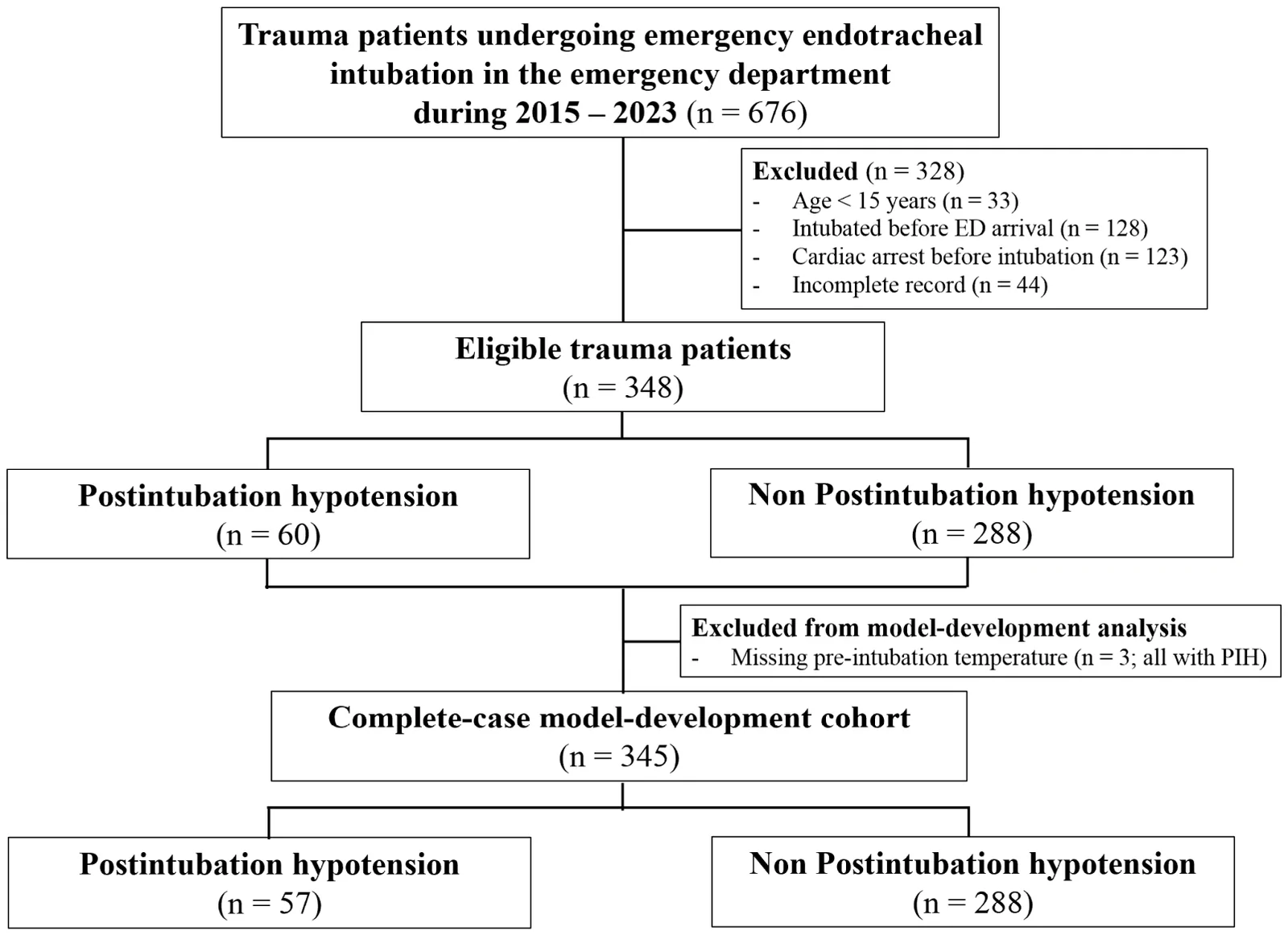
Study flow diagram. Of 348 eligible patients, 60 (17.2%) developed PIH. Exclusion of 3 patients with missing temperature left 345 complete cases for model development and internal validation. ED: emergency department; PIH: postintubation hypotension.

A total of 676 consecutive trauma patients were screened, of whom 348 met the eligibility criteria and were included in the study (Figure 1). PIH occurred in 60 patients (17.2%). Preintubation body temperature was missing in three patients (0.9%), all of whom developed PIH. Consequently, the complete-case cohort used for model development and internal validation comprised 345 patients, including 57 with PIH and 288 without PIH. Patient characteristics according to PIH status are presented in Table 1.

Patients who developed PIH had lower preintubation SBP, DBP, body temperature, peripheral oxygen saturation, GCS score, and hematocrit, and a higher heart rate than patients without PIH (Table 1). Patients with PIH also had a higher ISS, a greater prevalence of torso injury, and a greater number of injured anatomical regions. Respiratory rate and the distribution of injury mechanism did not differ significantly between groups.

### 3.2. Development of the PIH Prediction Model

Univariable associations between the eight candidate predictors and PIH are presented in Supplementary Table S1. Following backward elimination from the initial eight-predictor model, four preintubation variables were retained (Table 2): SBP, body temperature, hematocrit, and torso injury. Lower SBP was associated with higher odds of PIH (aOR 1.19 per 10-mmHg decrease, 95% CI 1.06–1.33; *p* = 0.004), as were lower body temperature (aOR 1.48 per 1 °C decrease, 95% CI 1.10–1.98; *p* = 0.010) and lower hematocrit (aOR 1.33 per 5-percentage-point decrease, 95% CI 1.07–1.66; *p* = 0.011). Torso injury was also associated with higher odds of PIH (aOR 3.26, 95% CI 1.55–6.88; *p* = 0.002).

The fitted model was as follows:

logit[P(PIH)] = 16.419 − 0.0171(SBP) − 0.3890(temperature) − 0.0575(hematocrit) + 1.1821(torso injury),

where SBP is expressed in mmHg, temperature in °C, and hematocrit as a percentage, with torso injury coded as 1 when present and 0 when absent.

### 3.3. Model Performance and Internal Validation

**Figure 2:**
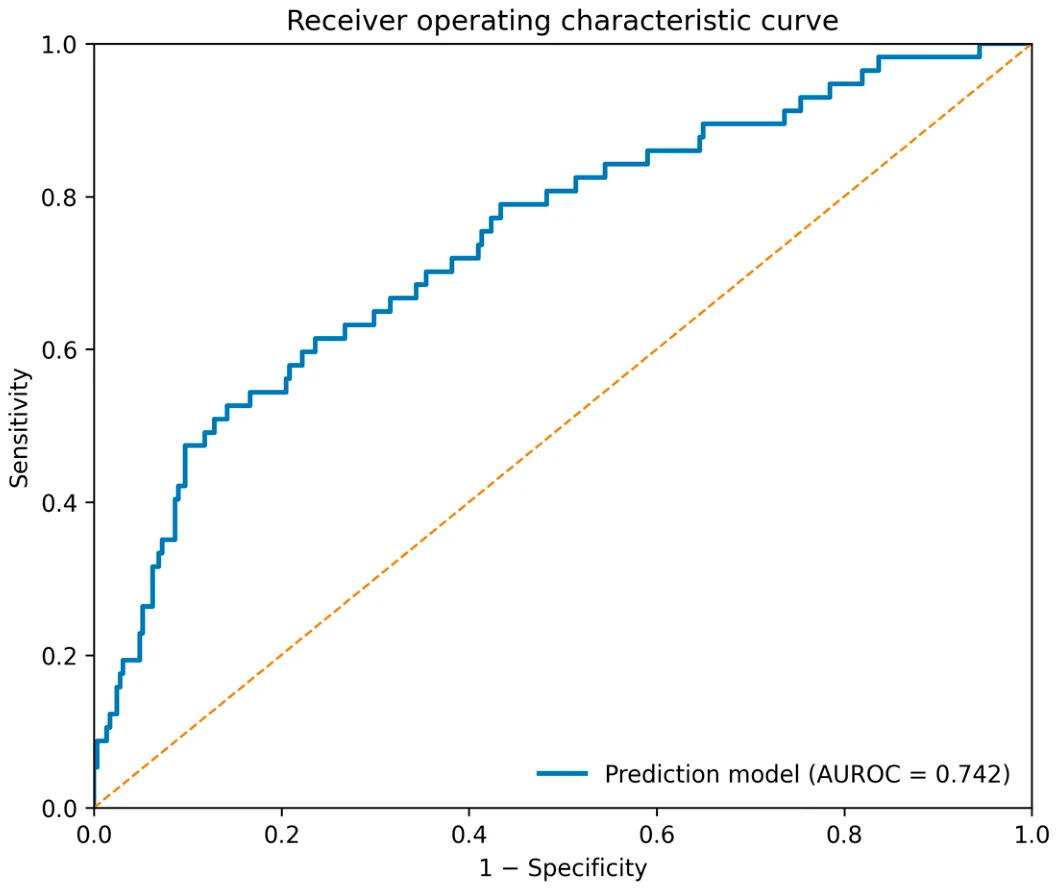
Receiver operating characteristic curve of the final four-predictor model. The apparent area under the receiver operating characteristic curve was 0.742. The dashed diagonal line represents perfect calibration.

**Figure 3:**
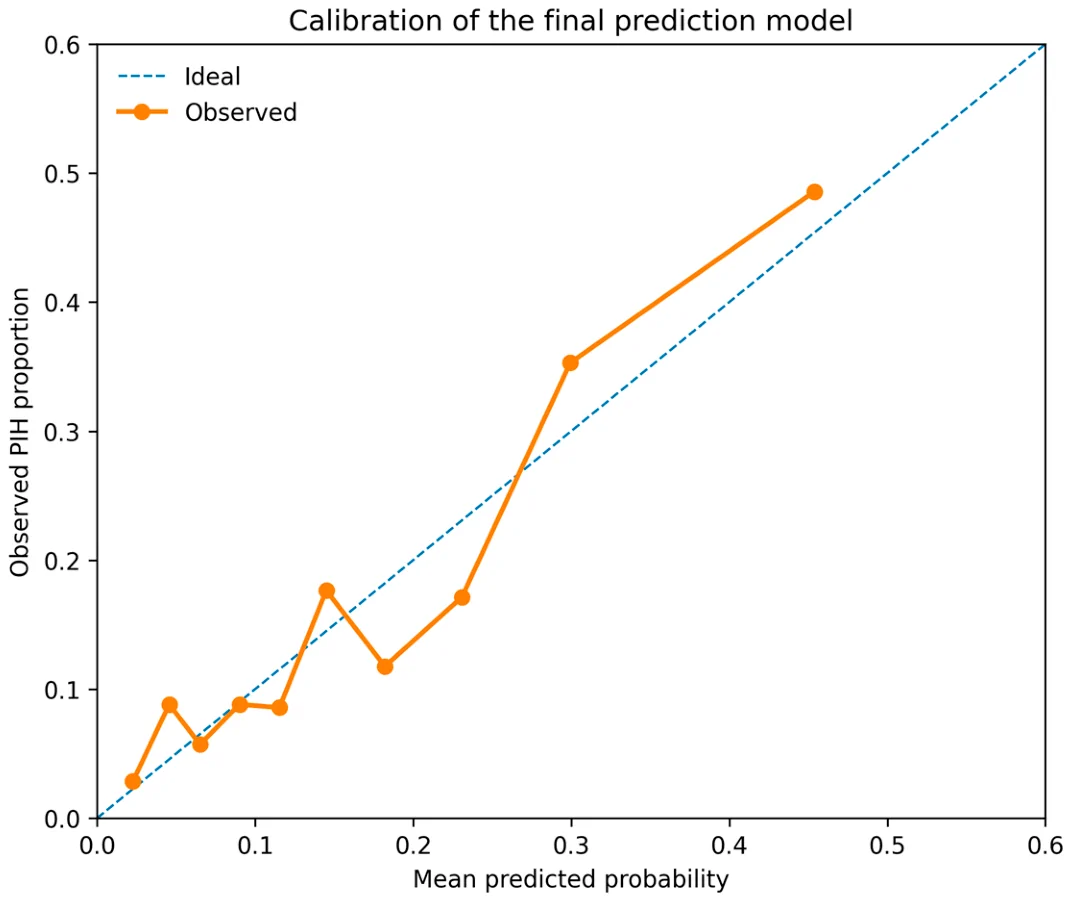
Apparent calibration of the final prediction model in the development cohort. Points represent the observed proportion of postintubation hypotension and the mean model-predicted probability within groups of predicted risk. The dashed diagonal line represents perfect calibration. This plot describes apparent calibration in the development data and does not represent external validation or optimism-corrected calibration.

The final model had an apparent AUROC of 0.742 (bootstrap 95% CI, 0.663–0.812) and apparent Brier score of 0.119 (Figure 2). After repeating the entire model-development procedure in 2000 bootstrap resamples, the estimated AUROC optimism was 0.043, resulting in an optimism-corrected AUROC of 0.700. The optimism-corrected Brier score was 0.127. The mean calibration slope of the bootstrap-derived models evaluated in the original development dataset was 0.80 (Figure 3).

### 3.4. Bootstrap Model Stability

**Figure 4:**
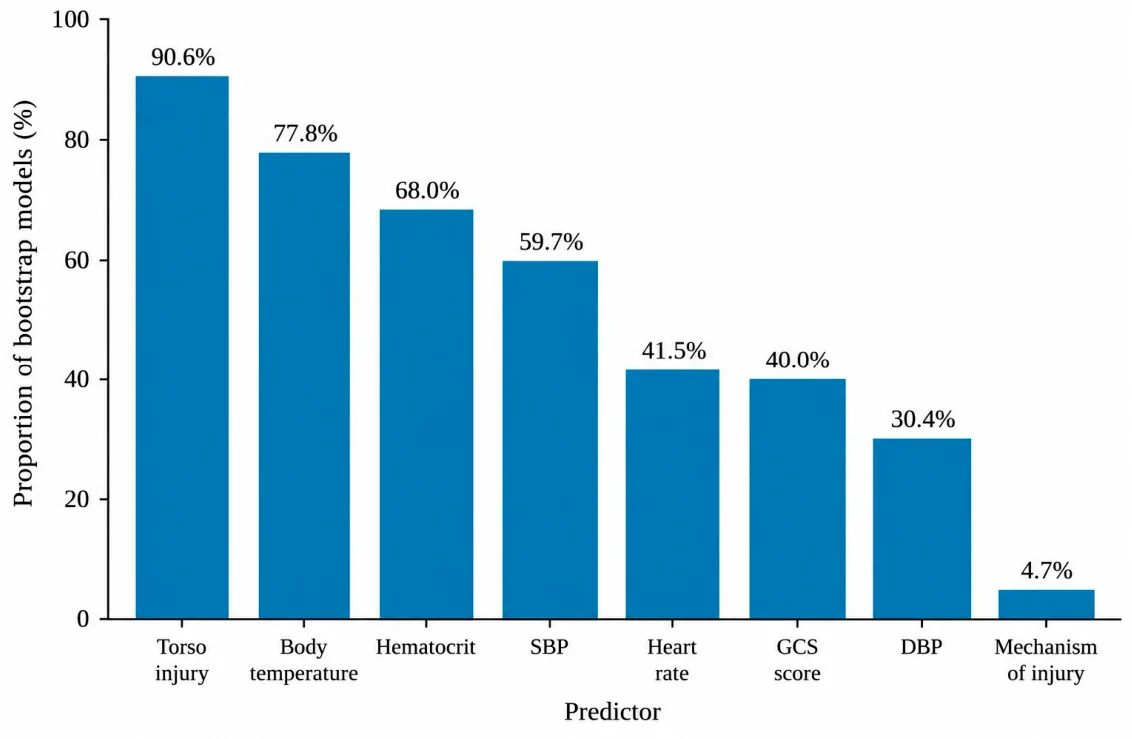
Predictor-selection stability during bootstrap internal validation. The complete backward-selection procedure was repeated within each bootstrap sample; percentages indicate the proportion of bootstrap models in which each candidate predictor was retained.

Predictor retention varied across bootstrap resamples (Figure 4). Torso injury was retained most frequently (90.6%), followed by body temperature (77.8%), hematocrit (68.0%), and SBP (59.7%). Heart rate, GCS score, DBP, and mechanism of injury were retained in 41.5%, 40.0%, 30.4%, and 4.7% of bootstrap models, respectively. The original four-predictor combination was reproduced in 13.4% of bootstrap models, indicating substantial model-selection uncertainty.

## 4. Discussion

### 4.1. Main Findings

This study developed and internally validated a preintubation prediction model for PIH in trauma patients undergoing emergency endotracheal intubation in the ED. The final model retained four preintubation characteristics: SBP, body temperature, hematocrit, and torso injury. The apparent AUROC was 0.742 and decreased to 0.700 after bootstrap correction that repeated the entire model-development procedure. This attenuation, together with the bootstrap-derived mean calibration slope of 0.80 and variability in predictor retention across resamples, suggests potential overfitting and model-selection uncertainty, limiting confidence in the stability and transportability of the model.

The retained predictors are clinically plausible markers of preintubation physiological vulnerability. Lower SBP may reflect reduced circulatory reserve and greater susceptibility to hemodynamic deterioration following induction and positive-pressure ventilation [11]. Lower body temperature may reflect shock severity, environmental exposure, or impaired thermoregulation in severely injured patients [12]. Lower hematocrit may be associated with blood loss or reduced oxygen-carrying capacity, although early hematocrit values in acute hemorrhage should be interpreted cautiously. Torso injury may serve as a readily identifiable marker of injury patterns associated with occult hemorrhage or compromised cardiopulmonary reserve [13]. Collectively, these variables represent potentially informative physiological and anatomical characteristics, although their selection and estimated effects require confirmation in independent cohorts.

The developed model focuses on information potentially available immediately before intubation. Accordingly, ISS and the number of injured anatomical regions were not included as candidate predictors because they require more comprehensive injury assessment and may not be reliably available at the intended preintubation prediction time point. Furthermore, continuous physiological predictors were retained on their original scales, avoiding the loss of information and artificial discontinuities associated with dichotomization.

### 4.2. Comparison with Previous Evidence

Previous studies have demonstrated associations between preintubation physiological characteristics and PIH. Heffner et al. reported that a shock index (SI) ≥ 0.8 predicted PIH with 67% sensitivity and 80% specificity [8], while Lee et al. found modest discrimination with AUROCs of 0.611, 0.676, and 0.614 for SI, age-adjusted SI, and modified SI in ED patients, respectively [9]. Trauma-specific prediction approaches have also been developed. Elmer et al. derived and validated a prehospital risk score in 4866 trauma patients [6], whereas Price et al. identified age, tachycardia, multisystem injury, and prehospital crystalloid administration as predictors of PIH in 998 patients undergoing prehospital emergency anesthesia [7]. However, differences in clinical settings, patient selection, predictor availability, airway management practices, and outcome definitions may limit the direct transportability of these approaches to trauma patients undergoing emergency endotracheal intubation in the ED. Importantly, differences in study populations and outcome definitions preclude direct comparisons of published discrimination estimates. Whether the present model offers incremental predictive value over simple clinical assessments, such as SBP or shock index alone, remains unknown and requires head-to-head evaluation in the same population.

This distinction may be particularly relevant for settings in which prehospital resources and capabilities vary. In Thailand, the availability of advanced prehospital care, trained personnel, and equipment varies across settings [14]. Risk prediction should also be considered within the broader context of peri-intubation resuscitation. Contemporary approaches emphasize circulatory optimization before intubation and strategies to minimize peri-intubation hemodynamic deterioration, including appropriate induction-agent selection, vasopressor support, and other resuscitative interventions [15,16,17,18,19,20]. Preintubation risk assessment may complement these measures by identifying patients who may benefit from greater attention to hemodynamic preparation before induction.

Against this background, the present study developed and internally validated a pragmatic PIH prediction model specifically for trauma patients undergoing emergency endotracheal intubation in the ED using preintubation variables. The model represents an exploratory approach to preintubation risk prediction and is not intended for clinical decision-making without further validation. External validation is required to assess its transportability, calibration, and potential clinical utility in other settings.

### 4.3. Clinical Interpretation and Implications

The model is consistent with the concept of physiology-first airway management, which emphasizes recognition and correction of physiological vulnerability before airway intervention [21,22,23]. Lower SBP, lower body temperature, lower hematocrit, and torso injury may represent complementary markers of physiological vulnerability and injury burden before intubation.

Importantly, the model’s intended clinical role is defined by its prediction time point. By incorporating only preintubation characteristics, it estimates baseline hemodynamic vulnerability before intubation rather than accounting for all factors influencing subsequent PIH. Peri-intubation management, including induction-agent selection and dosing, airway management, vasopressor administration, and ongoing resuscitation, may substantially modify the risk of hypotension. Consequently, the model cannot account for treatment-related variability or predict risk under different management strategies.

From a clinical perspective, if externally validated and shown to provide incremental clinical value, the model may help identify patients who could benefit from greater vigilance and attention to hemodynamic preparation before induction, including assessment of circulating volume and perfusion, vasopressor readiness, and selection of an induction strategy appropriate to the patient’s physiological condition [15,18,19,20]. However, predicted risk should complement, rather than replace, clinical assessment and established resuscitation practices and should not delay an urgently indicated airway intervention.

No clinical decision threshold was derived from this development cohort. Predicted probabilities should, therefore, not be interpreted as prescriptive cut-points for intervention. Any potential thresholds should be evaluated only after external validation and formal assessment of clinical utility. Notably, the optimism-corrected AUROC was 0.700, while bootstrap validation demonstrated substantial model-selection uncertainty. Thus, the model should not be used as a stand-alone clinical decision rule at this stage. External validation is required to assess its reproducibility, calibration, and clinical utility in independent trauma populations.

### 4.4. Strengths and Limitations

This study has several strengths. It included a consecutive cohort of trauma patients undergoing emergency endotracheal intubation in the ED over a 9-year period, reflecting routine emergency airway practice at a level I trauma center. The prediction model was restricted to information available before intubation, preserving the intended prediction time point and avoiding procedural or postintubation variables. Continuous physiological predictors were retained on their original scales to preserve predictive information. Internal validation repeated the entire model-development procedure within 2000 bootstrap resamples, accounting for optimism introduced by variable selection.

Several limitations should be acknowledged. First, the single-center retrospective design is susceptible to incomplete documentation, information bias, and residual confounding. Three patients with missing preintubation temperature data were excluded from model development, all of whom developed PIH. Variations in airway management and resuscitation practices over the 9-year period, including intubation without induction agents or neuromuscular blockade, may have influenced PIH and limit generalizability to settings using standardized RSI protocols. Procedural and treatment-related factors were excluded to preserve the preintubation prediction time point, potentially limiting the model’s ability to account for differences in peri-intubation management. Intra-abdominal hemorrhage could not be reliably confirmed in all patients, particularly those who died before definitive evaluation, preventing assessment of its specific contribution to PIH. Additionally, noninvasive blood pressure measurements at 15–30-min intervals may have missed transient hypotensive episodes.

Second, the limited number of PIH events relative to the eight candidate predictors increased the potential for overfitting. Bootstrap analysis demonstrated substantial model-selection instability, with the original four-predictor combination reproduced in only 13.4% of resamples. *p*-value-based backward elimination may also introduce selection bias and exaggerate coefficient estimates. Although bootstrap validation repeated the entire selection procedure to estimate optimism, it cannot eliminate this instability. These findings warrant cautious interpretation of individual predictor effects and support evaluation in larger cohorts using more robust approaches, such as penalized regression.

Third, model performance decreased after optimism correction, and internal validation alone cannot establish performance in independent populations. The model has not been externally validated, directly compared with existing prediction approaches in the same population, or evaluated for clinical net benefit. Although temperature was routinely measured during ED triage and blood samples for hematocrit were generally collected during initial resuscitation, hematocrit results may not have been available before intubation in all patients. Similarly, torso injury identified retrospectively may not have been recognized before intubation. These constraints may limit the feasibility of real-time model applications.

## 5. Conclusions

This exploratory study developed and internally validated a four-variable preintubation prediction model for postintubation hypotension in trauma patients undergoing emergency endotracheal intubation. The model demonstrated modest optimism-corrected discrimination and substantial predictor-selection instability, limiting confidence in its reproducibility and transportability. Although the identified predictors may reflect preintubation physiological vulnerability in this cohort, the model is not suitable for routine clinical decision-making at present. External validation, prospective assessment of predictor availability, and evaluation of incremental clinical utility are required before implementation.

## Figures and Tables

**Table 1:** Characteristics of the study population according to postintubation hypotension status.

Characteristics	PIH (*n* = 60)	Non-PIH (*n* = 288)	*p*-Value
**Demographic characteristics**			
Age (years), median (IQR)	36.5 (25.5–61)	37 (24–58)	0.553
Male sex	49 (81.7)	224 (77.8)	0.606
BMI (kg/m^2^), mean ± SD	23.09 ± 3.59	23.43 ± 3.40	0.478
**Preintubation physiological characteristics**			
SBP (mmHg), mean ± SD	134.1 ± 26.7	150.7 ± 31.0	<0.001
DBP (mmHg), mean ± SD	86.4 ± 24.6	93.7 ± 24.0	0.035
HR (beats/min), mean ± SD	106.1 ± 27.8	97.3 ± 24.5	0.014
RR (breaths/min), mean ± SD	25.9 ± 8.9	25.2 ± 7.6	0.546
Body temperature (°C), mean ± SD *	36.18 ± 1.25	36.68 ± 1.00	0.001
SpO_2_ (%), median (IQR)	93.5 (83–99)	97 (94–100)	<0.001
GCS, median (IQR)	7 (3–13)	8 (6–11.5)	0.025
Hematocrit (%), mean ± SD	38.2 ± 7.0	40.7 ± 6.4	0.007
**Injury characteristics**			
Injury Severity Score, median (IQR)	35 (25–46.5)	20 (14–30)	<0.001
Torso injury	50 (83.3)	163 (56.6)	<0.001
Number of injured regions, median (IQR)	3 (2–4)	2 (1–3)	<0.001
**Mechanism of injury**			0.101
Blunt trauma	57 (95.0)	258 (89.6)	
Penetrating trauma	3 (5.0)	8 (2.8)	
Burn	0	19 (6.6)	
Blast injury	0	3 (1.0)	

* indicates that preintubation body temperature was available for 57 patients with PIH and all 288 patients without PIH. Bold text indicates variable category headings. Data are presented as numbers (%) unless otherwise indicated. IQR: interquartile range; BMI: body mass index; GCS: Glasgow Coma Scale; SD: standard deviation; SBP: systolic blood pressure; DBP: diastolic blood pressure; HR: heart rate; RR: respiratory rate; SpO_2_: oxygen saturation.

**Table 2:** Final multivariable logistic regression model for postintubation hypotension.

Predictor	Effect Scale	Adjusted OR	95% CI	*p*-Value
Preintubation SBP	per 10-mmHg decrease	1.19	1.06–1.33	0.004
Body temperature	per 1 °C decrease	1.48	1.10–1.98	0.010
Hematocrit	per 5-point decrease	1.33	1.07–1.66	0.011
Torso injury	present vs. absent	3.26	1.55–6.88	0.002

CI: confidence interval; OR: odds ratio. Odds ratios for continuous predictors are presented per clinically interpretable decrease as specified.

## Data Availability

All the data created or analyzed during this work are available from the corresponding author upon reasonable request.

## Supplementary Materials

The following supporting information can be downloaded at: https://www.mdpi.com/article/10.3390/jcm15208102/s1. Table S1: Univariable logistic regression analysis of candidate predictors for postintubation hypotension.

## Abbreviations

The following abbreviations are used in this manuscript:

aOR	adjusted odds ratio
AUROC	area under the receiver operating characteristic curve
BMI	body mass index
CI	confidence interval
DBP	diastolic blood pressure
ED	emergency department
HR	heart rate
IQR	interquartile range
ISS	injury severity score
PIH	postintubation hypotension
RR	respiratory rate
SBP	systolic blood pressure
