# Supplementary material for: Development and Internal Validation of a Preintubation Prediction Model for Postintubation Hypotension in Trauma Patients Undergoing Emergency Endotracheal Intubation: A Retrospective Cohort Study
Source: J Clin Med. 2026 Oct 19;15(20):8102. doi: 10.3390/jcm15208102 (PMC13706554; doi:10.3390/jcm15208102)
Supplement: Supplementary file 1 [file jcm-15-08102-s001.zip › jcm-4637351-supplementary.pdf]

Supplementary Table S1. Univariable logistic regression analysis of candidate predictors for postintubation hypotension

Candidate predictor	Effect scale	Unadjusted OR	95% CI	P-value
Pre-intubation SBP	per 10-mmHg decrease	1.23	1.10–1.38	<0.001
Pre-intubation DBP	per 10-mmHg decrease	1.17	1.03–1.34	0.019
Heart rate	per 10-beats/min increase	1.12	1.00–1.25	0.055
Body temperature	per 1 °C decrease	1.60	1.20–2.12	0.001
Glasgow Coma Scale	per 1-point decrease	1.07	1.00–1.16	0.062
Hematocrit	per 5-percentage-point decrease	1.36	1.10–1.67	0.004
Blunt mechanism	blunt vs non-blunt	2.09	0.62–7.11	0.236
Torso injury	present vs absent	3.60	1.75–7.41	<0.001

Analysis was performed in the complete-case model-development cohort (n = 345; 57 PIH events).

Abbreviations: PIH, post-intubation hypotension; SBP, systolic blood pressure; DBP, diastolic blood pressure; GCS, Glasgow Coma Scale; OR, odds ratio; CI, confidence interval.

Note: Odds ratios are from separate univariable logistic regression models. Continuous predictors were modeled on their original scales and are presented using clinically interpretable effect scales. Mechanism of injury was modeled as blunt versus non-blunt trauma, and torso injury as present versus absent. Missing data were not imputed.
